# Comparison of Strategies to Detect Epistasis from eQTL Data

**DOI:** 10.1371/journal.pone.0028415

**Published:** 2011-12-19

**Authors:** Karen Kapur, Thierry Schüpbach, Ioannis Xenarios, Zoltán Kutalik, Sven Bergmann

**Affiliations:** 1 Department of Medical Genetics, University of Lausanne, Lausanne, Switzerland; 2 Swiss Institute of Bioinformatics, Lausanne, Switzerland; 3 Vital IT Group, Swiss Institute of Bioinformatics, Lausanne, Switzerland; University of Texas School of Public Health, United States of America

## Abstract

Genome-wide association studies have been instrumental in identifying genetic variants associated with complex traits such as human disease or gene expression phenotypes. It has been proposed that extending existing analysis methods by considering interactions between pairs of loci may uncover additional genetic effects. However, the large number of possible two-marker tests presents significant computational and statistical challenges. Although several strategies to detect epistasis effects have been proposed and tested for specific phenotypes, so far there has been no systematic attempt to compare their performance using real data. We made use of thousands of gene expression traits from linkage and eQTL studies, to compare the performance of different strategies. We found that using information from marginal associations between markers and phenotypes to detect epistatic effects yielded a lower false discovery rate (FDR) than a strategy solely using biological annotation in yeast, whereas results from human data were inconclusive. For future studies whose aim is to discover epistatic effects, we recommend incorporating information about marginal associations between SNPs and phenotypes instead of relying solely on biological annotation. Improved methods to discover epistatic effects will result in a more complete understanding of complex genetic effects.

## Introduction

Genome-wide association studies have been instrumental in identifying genetic variants associated with complex traits such as human disease or gene expression phenotypes [1]. However, for many human traits genetic variants discovered so far account for only 5–10% of the phenotypic variance [2]. For linkage studies in yeast, which are grown in tightly controlled environments, the explained variance of highly heritable gene expression traits is also limited, with one study reporting a median explained variance of 27% and finding no associated genetic variants for many (40%) gene expression traits [3]. The challenge of identifying additional genetic variants which explain a larger proportion of phenotypic variance is of great interest and has been coined the problem of the missing heritability [4]. One avenue to discover additional genetic effects is to consider epistasis, i.e. joint effects between markers. While many studies have analyzed effects of individual markers, only recently have studies begun to extend analysis methods to consider interaction effects between pairs of loci [5]–[10].

Due to the large number of two-marker models that need to be evaluated, searching for epistatic effects poses both computational and statistical challenges. Whereas many human genome-wide association studies test on the order of one million SNPs, considering all pairs of SNPs amounts to approximately 500 billion tests, since the number of pairs of SNPs scales quadratically with the number of markers. The large number of tests to perform incurs a considerable computational burden, although this challenge is being increasingly addressed. Computational solutions include parallelizing the computations [11], graphics hardware based computing [12], and implementing approximations for case-control studies [13]. Therefore, ultimately the most pressing problem is how to handle the statistical issue of multiple testing. As a result of performing so many tests, a very stringent type I error threshold is needed to prevent selection of false positives. However, at such a threshold many true positives are being missed. In addition, epistatic tests have a complex dependency structure, which requires cumbersome permutation procedures in order to properly assess the type I error rate.

The multiple testing problem has been addressed in previous expression quantitative trait locus (eQTL) studies by reducing the number of SNPs tested for association with gene expression phenotypes. To search for genetic variants which are associated with a particular gene expression level, many researchers have restricted the search to those variants proximal to the gene in question, knowns as *cis* eQTLs [14], [15]. In contrast, to search for non-proximal variants associated with gene expression (*trans* eQTLs), approaches may select SNPs hypothesized as more likely to affect gene expression such as non-synonymous SNPs, *cis* associated SNPs to any gene, or splicing SNPs [14]. Other methods weight genetic variants based on their regulatory features, incorporating information such as gene function, conservation, position and type of genetic polymorphisms [16]. In vitro information about the DNA-binding specificity of a transcription factor can be included to map loci [17]. Moreover, integration of multiple data types has been suggested to aid in detecting and interpreting regulatory variants [18], [19].

Similar methods have been proposed to reduce the number of SNP pairs to detect epistasis. A marginal-by-genomewide approach was proposed, in which SNP pairs are chosen such that at least one SNP has a relatively strong marginal association with the trait [20], [21]. Other approaches have used protein-protein interaction information from STRING [22] to prioritize SNP pairs [9] and CNV pairs [23]. Another paper proposed using either disease-dependent information based on previously detected associations or using existing biological databases to define candidate SNP pairs to test for epistasis [10]. Additionally, a specific biological hypothesis, such as the interaction between regulatory and protein-coding variants [24], can drive an approach to studying epistatic effects.

While many strategies to detect epistasis effects have been proposed and tested for specific phenotypes, so far there has been no systematic attempt to compare their performance using real data. In this work we present a comparative analysis of various strategies. To evaluate any approach we first sought for a measure of performance, identifying the false discovery rate (FDR) as the most relevant indicator. The immense computational burden was overcome using optimized computations and massive parallel computing [11] on a large computer cluster (www.vital-it.ch). In terms of data, it is clear that when considering a single trait it is difficult to assess which strategy for selecting SNP pairs may be optimal. Therefore we made use of gene expression traits generated in linkage and eQTL studies, since these data include thousands of traits, of which a large fraction is known to have a strong genetic component. Specifically, we first analyzed data from a yeast linkage study [25], encouraged by reports from several studies identifying interacting loci [3], [26] and the smaller complexity compared to human data. We found that information from marginal associations is more informative than using STRING annotations. However, using the data from a human eQTL study, we did not find convincing evidence for systematically improved performance of strategies relying on marginal associations or gene annotations. Although we had considerable computational resources at our disposal, we were faced with considerable study limitations. Nevertheless, we found several putative associations when testing a small number of SNPs with strong marginal effects.

## Results

We first used data from a yeast linkage study, consisting of 112 segregants derived from a cross of a yeast lab strain (S288C) and a wild isolate, as described in [25], to study epistatic effects. We initially attempted a naïve method to detect epistatic effects which searches over all possible SNP pairs. For 2,931 markers, there were a total of 4,293,915 SNP pairs. Using 10,000 permutations, at cutoffs defined by the 1% and 0.1% quantile of the permutation statistics (see Methods), we estimated an FDR of 96% and 99%, respectively. The number of SNP pairs selected at these thresholds closely matches the number of SNP pairs expected by chance.

Next, we tried several different strategies to reduce the number of SNP pairs under consideration, marginal-by-marginal (MM), marginal-by-genomewide (MG) and STRING (ST) strategies. We test the epistasis model (see Methods) using a subset of SNP pairs defined by each strategy. The MM strategy selects a set of SNP pairs such that both SNPs in the pair are associated with the trait at a given significance level, determined from the one-dimensional regression model (see Methods). The MG strategy selects a set of SNP pairs such that at least one SNP in the pair is associated with the trait at a given significance level. The ST strategy selects pairs of genes with corresponding protein-protein interactions in the STRING database determined by a given significance threshold. The strategy includes all SNP pairs that map to a gene pair.

**Figure 1:**
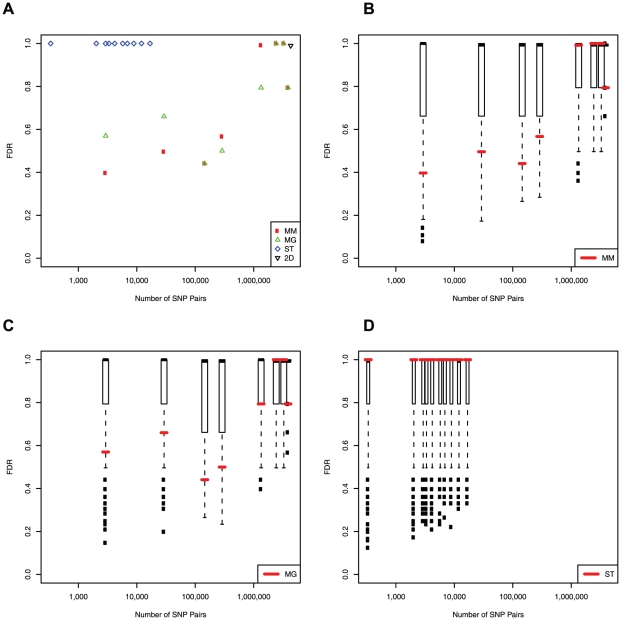
Comparison of the FDR (determined at cutoffs corresponding to the 0.1% quantile of permutation p-values) for detecting interactions in yeast gene expression data among the different subset strategies. (A) The FDR is plotted against the number of SNP pairs for MM, MG and ST in red, green and blue, respectively. (B–D) The FDR is shown for MM, MG and ST strategies compared to 500 MM^0^, MG^0^ and ST^0^ control strategies, respectively. Significance values are computed as the proportion of control strategies with FDR as low or lower. (B) Significance values 0.052, 0.072, 0.088, 0.05, 0.16, 0.54, 1.0, 1.0 and 0.15. (C) Significance values 0.17, 0.17, 0.048, 0.13, 0.32, 1.0, 1.0 and 0.16. (D) Significance values 1.0, 1.0, 1.0, 1.0, 1.0, 1.0, 1.0, 1.0, 1.0, 1.0.

We compared the different strategies by comparing the estimated FDR as shown in Figure 1A. We define a p-value cutoff by the 0.1% quantile of the permutation statistics which fixes the expected number of false positives. We found that overall the MM and MG strategies tend to have lower FDRs than the ST strategy. We can also observe that for the MM and MG strategies, as the number of tests increases, the FDR tends to increase. This trend is due to the statistical issue of multiple testing, reflecting that as the number of tests increases it becomes difficult to distinguish the significance of true interaction effects from those expected by chance. Indeed, we found that selecting a very small number of marginal SNPs gives the smallest FDR.

**Figure 2:**
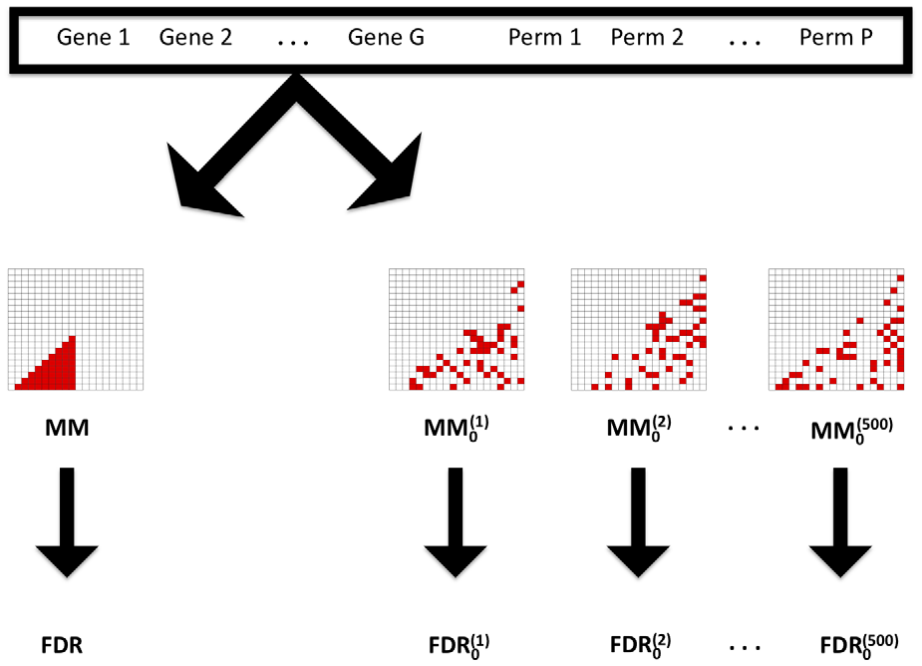
Illustration of comparisons between subset strategies and control strategies for the MM strategy. A subset strategy is applied to both gene expression measurements and randomly permuted measurements. The gene expression measurements define the number of hits at a given p-value threshold while the permutations are used to estimate the expected number of false positives, giving rise to an estimate of the FDR.

We also compared the strategies by comparing their performance to an appropriate control strategy (MM^0^, MG^0^ and ST^0^, respectively; see Methods and Figure 2 for further details). The goal is to assess whether the information in the candidate strategies aids in the detection of epistatic effects. The performance relative to 500 randomly chosen respective controls is given in Figure 1B–D. Both the MM and MG strategies result in a lower FDR than the random control (p = 0.07, p = 0.17, respectively for approximately 3,000 tests). In contrast, the ST strategy does not outperform the random control at any significance threshold (p = 1.0). The FDR as a function of the p-value cutoff is shown in Figure S1.

**Figure 3:**
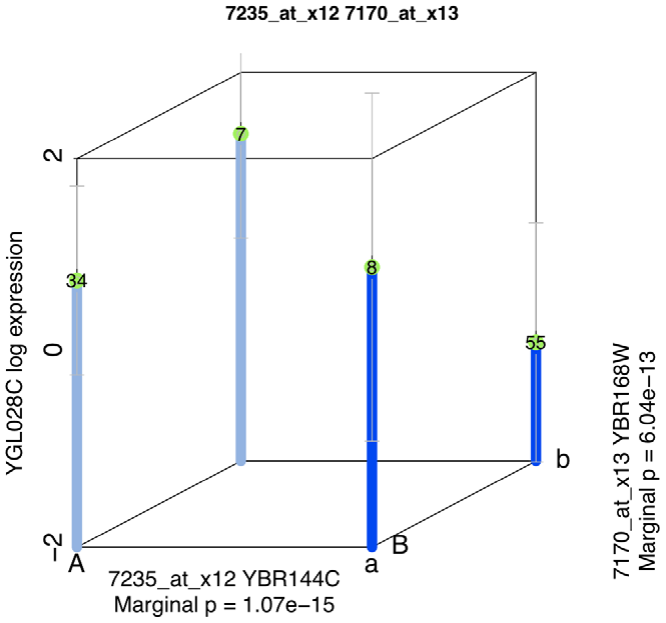
The data supporting the most significant interaction from the MM strategy is shown here. Capital letter markers refer to RM11; lowercase letter markers refer to BY4716 (S288c). Blue bars mark the model predicted expression levels at each combination of genetic markers, green dots show the observed mean expression levels, and grey bars show the standard deviation.

The best performing strategy was the MM strategy. The FDR was 39.7%, with 10 hits selected at this cutoff, whereas the expected number of false positives was below four. A plot of the most significant interaction from the MM strategy is given in Figure 3.

We then tried a similar approach to systematically compare the performance of different strategies for human data. However, we were faced with considerable study limitations, including larger computational and statistical complexity and stronger environmental effects. We chose a restricted set of 297,153 HapMap SNPs (see Methods), corresponding to 44 billion possible SNP pairs. As in yeast, we performed permutation tests to assess the significance of the interaction test statistics. However, even with our large computational resources we were only able to perform 1,000 permutations. With fewer permutations, we are required to use a less stringent p-value threshold to assess significance of the results. As a result, we had less power to separate out the strongest signals from noise. We note that in the analysis of yeast, using the same (1%) quantile cut-off as for the human data (instead of the 0.1% quantile), we do not observe improved performance of the MM or MG strategies compared to the ST strategy, with all strategies giving FDRs of 78–100%.

Applying the naïve method to detect epistatic effects which searches over all possible SNP pairs, at a p-value cutoff defined by the 1% quantile of the permutation statistics, we find an FDR of 82%. Thus, of the 12 results selected, only about two are expected to be true.

**Figure 4:**
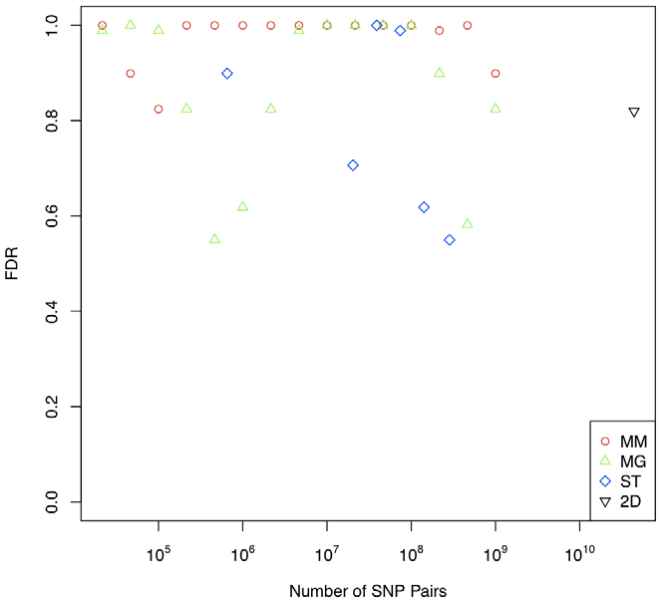
Comparison of the FDR (determined at cutoffs corresponding to the 0.1% quantile of permutation p-values) for detecting interactions in human gene expression data among the different subset strategies. The FDR is plotted against the number of SNP pairs for MM, MG and ST in red, green and blue, respectively.

Next, we compared the performance of MM, MG and ST strategies. As shown in Figure 4, we do not see any trend in performance suggesting that any of the MM, MG or ST strategies achieve superior performance.

In order to facilitate the computation of a larger number of permutations, we decided to evaluate the MM strategy using a very limited set of SNPs. For such a small set of SNPs it was therefore possible to perform 10,000 permutations. We applied the MM strategy using the top 5,000 marginally significant SNPs for each gene expression trait, corresponding to 1.2 million SNP pairs. We assessed the significance using 10,000 permutation tests. At the 0.1% quantile of the permutation statistics we estimate an FDR of 0.33, detecting three putative hits of which approximately one is expected by chance (Figure S2).

Details of the genes and SNPs associated epistatically are given in Table 1. A plot of the FDR at different p-value thresholds is given in Figure S3. We tested the epistatic association with expression levels of HLA-DRB1 in other populations obtaining p-values of 0.0055, 0.0098, 0.015, 0.00012, 0.93, 0.40, 0.59 for CHB, JPT, GIH, MEX, LWK, MKK and YRI, respectively, indicating that the interaction replicates well across non-African populations (see also Figure S4). We estimate the percent variance explained by the epistasis term to be 9.3%, 7.9%, 7.0%, and 28.7% in CHB, JPT, GIH, and MEX, respectively. We asked whether the interaction effect for HLA-DRB1 would disappear if we take into account dominant or recessive effects. We found that the interaction remains significant (p = 1.631e-11, CEU). Both SNPs fall within CNV regions based on the Database for Genomic Variants [27]. However, the Hardy-Weinberg p-values are not significant (p = 0.575, p = 0.646) indicating that these SNPs are not likely to fall into the copy number variant region.

**Table 1:** Details of the two hits discovered by the MM strategy in a human CEU eQTL dataset.

Gene	Probe	Snp1	Snp2	P-value
HLA-DRB1	ILMN_20550_7330093	rs3763313	rs3129883	1.79e-14
HLA-DRB5	ILMN_3178_4390692	rs984778	rs206017	1.19e-13
IFIT3	ILMN_1944_2690452	rs2197025	rs2031339	9.13e-12

The FDR is estimated at 49%.

## Discussion

We compared several strategies to derive a subset of candidate SNP pairs to test for epistatic effects. We found that, in yeast, strategies making use of marginal association information outperform the STRING strategy which relies solely on annotations of protein-protein interactions. We found that the MM strategy can give rise to a set of epistatic effects with a lower proportion of false positives than naïvely testing all possible SNP pairs. This fact indicates that SNP pairs with the largest marginal associations tend to be enriched for epistatic effects.

In our comparison among the different strategies in human, we did not find evidence that one strategy outperforms another. However, our ability to differentiate between the strategies has several limitations. The larger number of SNPs to consider in human compared to yeast significantly increased the computational time (indeed we required several thousand CPU days on the high-performance computing facility VITAL-IT (http://www.vital-it.ch)). We examined only a subset of SNPs and we were only able to perform 1,000 permutations for the permutation test whereas we used 10,000 permutations in yeast. Furthermore, a dataset with larger number of individuals would provide more power in the multiple testing setting to detect epistatic effects.

Applying the MM strategy to a limited set of SNPs, we were able to detect one interaction in the CEU data which replicated in several non-African populations. Therefore, this finding likely represents a true interaction. A pair of SNPs near the HLA-DRA gene showed an epistatic effect on the expression levels of HLA-DRB1. Furthermore, including the epistatic relationship in the model of genetic effects on HLA-DRB1 expression increases the percent of explained variance by 7–28%.

However, the MM strategy did not result in many positive findings. The limited success may be due to the increased complexity of human data relative to yeast or perhaps due to smaller marginal effects in human than in yeast. Environmental variation may also play a large role for humans whereas the yeast strains were grown in a tightly controlled environment. Future studies, particularly with larger sample sizes, would be worthwhile to determine the limitations of the MM strategy.

One of the drawbacks to the MM strategy is that by definition it can only identify interactions between SNPs with marginal associations, while it misses non-marginally associated SNPs which may have epistatic associations. In contrast, the MG approach in principle allows for identification of new SNPs involved in genetic associations through epistasis, as long as this interaction involved another SNP with a statistically significant marginal association. Thus the fact that the performance of MM and MG are not very different, would perhaps point to MG as the best compromise between power and the ability to identify novel interesting loci.

Although we found that marginal associations are more informative for identifying epistasis than information from the STRING database, future approaches may benefit from incorporating biological information in more sophisticated ways. Using knowledge of transcription factors which regulate the gene's expression patterns, weighting SNPs based on their properties (i.e. synonymous, non-synonymous, cross-species conservation score) or using mutual information from several markers at once for case-control studies [28] may also prove worthwhile.

## Methods

### Data

#### Yeast

The yeast data were taken from 112 segregants derived from a cross of a yeast lab strain (S288C) and a wild isolate, as described in [25].

Gene expression measurements were subjected to the following exclusions and pre-processing. We excluded genes not included in the list provided by [29]. We required the gene expression values to have at least 90% non-missing values across the segregants. We normalized the data by subtracting the mean value calculated across all spots on the array and averaged the gene expression from the dye-swapped experiments. Log gene expression values were normal quantile transformed. Missing values were set to zero. The transformation to normal distribution was done for computational ease for permutation testing (see below). A total of 3,970 genes were included in our analysis.

We used genotype measurements from all 2,931 quality-controlled markers as described in [25].

#### Human

We used genotypic data from several HapMap populations (CEU, n = 109; CHB, n = 80; GIH, n = 82; JPT, n = 82; LWK, n = 83; MEX, n = 45; MKK, n = 138; YRI, n = 108) [30] in combination with expression profiles for lymphoblastoid cell lines generated from HapMap participants (commercial source: Coriell). Gene expression transcript levels were measured using Illumina's commercial whole genome expression array, Sentrix Human-6 Expression BeadChip version 2 (E. Dermitzakis, personal communication). We focused on data from the CEU panel since it has been well studied and many *cis* and *trans* effects have been reported [14].

We studied a subset of CEU candidate genes selected to have expression above background levels (mean transcript expression above average), high variation in expression (standard deviation ≥0.5) and a moderate number of genetic associations, using log transformed expression values. For each gene, we counted the number of marginal associations across the genome with p-values below 10^−5^. We included genes with at least the average number of such associations. The selection resulted in 989 gene expression probes.

The genotypes consist of 1.2 million HapMap SNPs. For the comparison of multiple strategies across different numbers of SNP pairs, we selected a subset of SNPs which uniquely mapped to gene regions and required minor allele frequency >0.20 (N = 297,153, see below). For the MM analysis (see Subset Selection Strategies below) we only required a minor allele frequency >0.05 (N = 1,223,296).

### Evaluation of Subset Strategies

A subset strategy defines a subset of SNP pairs which are tested for epistatic associations with a given set of phenotypes. For any given strategy we ask how many gene expression traits have at least one significantly associated epistasis effect. Hence, for each gene expression trait, it suffices to consider the minimum p-value across a SNP pair subset.

We assess the significance of the minimum p-value through permutation tests. Each permutation test consists of randomly permuting the phenotype values. We transform each gene expression phenotype via the normal quantile transformation so that all phenotypes have the same distribution. Therefore, a single set of permutations can be used to compare with all gene expression traits.

For any subset strategy we compare results from measured gene expression to results from permuted phenotypes. We chose a p-value cutoff in order to fix the expected number of false positives under the null hypothesis that no interaction effects exist. We then calculated the FDR, as used in [14]. We found this method to be useful to assess moderate FDR values, since no hits would be found for very stringent FDR cutoffs (such as 0.05). For yeast, we chose a p-value cutoff determined from the 0.1% quantile of the permutation values, using 10,000 permutations. This corresponds to the 10^th^ smallest of the 10,000 p-values. For the strategy comparison in human, due to computational considerations, we performed only 1,000 permutations and chose a p-value cutoff determined from the 1% quantile of the permutation values, corresponding to the 10^th^ smallest of 1,000 p-values.

### Subset Strategies

The marginal–by-marginal (MM) strategy selects a subset of SNP pairs such that both SNPs have marginal effects at a given significance threshold. The marginal-by-genomewide (MG) strategy selects a subset of SNP pairs such that at least one SNP has a marginal effect at a given significance threshold. The STRING (ST) strategy selects pairs of genes from the STRING database with scores above a given threshold. Larger scores indicate stronger evidence of a physical interaction between the corresponding proteins. STRING protein pairs are mapped to gene pairs which are subsequently mapped to corresponding SNP pairs.

For a SNP pair subset strategy, we define an appropriate control strategy (MM^0^, MG^0^ and ST^0^, respectively) whose aim is to ignore information used in the strategy's selection of SNP pairs. Both MM^0^ and MG^0^ strategies use marginally associated SNPs to a random phenotype. The MM^0^ strategy consists of SNP pairs such that both SNPs are marginally associated with the random phenotype. The MG^0^ strategy selects SNP pairs such that at least one SNP is marginally associated with the random phenotype. The ST^0^ strategy randomly chooses gene pairs such that both genes belong to the STRING database but are not necessarily connected in STRING. The gene pairs are subsequently mapped to corresponding SNP pairs.

The performance of the control strategy is evaluated on both the measured gene expression data and the permuted data, and the performance is compared between the two to estimate the FDR. In yeast, we carried out 500 analyses using the control.

### Mapping SNPs to Genes

In yeast we used the annotation provided by [25] to map SNPs directly to genes. We do not map upstream or downstream SNPs to the genes, but these SNPs are considered indirectly due to the strong linkage disequilibrium with surrounding markers arising from the linkage study. Note that only a subset of SNPs map to genes. Therefore we performed the STRING strategy on a smaller SNP subset than the MM and MG strategies. MM and MG strategies were also performed on the restricted subset of SNPs that map to genes in the STRING database (see Figure S5).

For the human data we mapped SNPs to genes provided they fell within 1 kb upstream or downstream of the transcription start site or within the gene region. We excluded SNPs mapping to multiple genes (N = 3,169) to enable easier mapping from STRING gene pairs to a set of SNP pairs.

### Tests of Genetic Association

Genetic association tests were carried out for marginal and epistatic models. The marginal model associates a phenotype, *y*, with the value of a single SNP, *x*. For haploid yeast genotypes *x* takes the value of either 0 or 1, whereas for diploid human genotypes *x* takes the value of 0, 1 or 2. We employ a simple normal linear model, 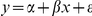, and assess the association between a SNP and a phenotype based on the significance of the β term. Marginal tests were carried out using PLINK [31].

The epistasis model includes effects of two SNPs, *x_1_* and *x_2_*. Again we use a normal linear model 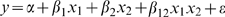. We assess the epistatic association between a pair of SNPs and a phenotype based on the significance of the β_12_ term. Epistasis tests were carried out using the FastEpistasis software [11].

### Ethics Approval

The research was conducted using HapMap cell lines which are a public resource, so no ethics approval was needed.

## Supporting Information

Figure S1 We plot the FDR for different p-value cutoffs, starting from the 0.1% quantile for the MM, MG and ST strategies separately.
(PDF)

Figure S2 Shown here is the fit of the epistasis model for the three interactions detected by the MM strategy, using 10,000 permutations. Gene expression phenotypes (A) HLA-DRB1, (B) HLA-DRB5 and (C) IFIT3.
(EPS)

Figure S3 We plot the FDR results from the MM strategy (top 5000 marginally associated SNPs) for different p-value cutoffs, starting from the 0.1% quantile.
(PDF)

Figure S4 Shown here is the fit of the epistasis model for the top interaction detected by the MM strategy, using 10,000 permutations. The fit is shown for several populations for which the effect replicated (A) CHB, (B) JPT, (C) GIH and (D) MEX.
(EPS)

Figure S5 Comparison of the FDR (determined at cutoffs corresponding to the 0.1% quantile of permutation p-values) for detecting interactions in yeast gene expression data among the different subset strategies. The analysis is restricted to SNPs mapping to genes in STRING. The FDR is plotted against the number of SNP pairs for MM, MG and ST in red, green and blue, respectively.
(PDF)

## References

[1] Hirschhorn JN, Daly MJ (2005). Genome-wide association studies for common diseases and complex traits.. Nat Rev Genet.

[2] Lango Allen H, Estrada K, Lettre G, Berndt SI, Weedon MN (2010). Hundreds of variants clustered in genomic loci and biological pathways affect human height.. Nature.

[3] Brem RB, Kruglyak L (2005). The landscape of genetic complexity across 5,700 gene expression traits in yeast.. Proc Natl Acad Sci U S A.

[4] Maher B (2008). Personal genomes: The case of the missing heritability.. Nature.

[5] Cordell HJ (2009). Detecting gene-gene interactions that underlie human diseases.. Nat Rev Genet.

[6] Curtis D (2007). Allelic association studies of genome wide association data can reveal errors in marker position assignments.. BMC Genet.

[7] Gayan J, Gonzalez-Perez A, Bermudo F, Saez ME, Royo JL (2008). A method for detecting epistasis in genome-wide studies using case-control multi-locus association analysis.. BMC Genomics.

[8] Herold C, Steffens M, Brockschmidt FF, Baur MP, Becker T (2009). INTERSNP: genome-wide interaction analysis guided by a priori information.. Bioinformatics.

[9] Emily M, Mailund T, Hein J, Schauser L, Schierup MH (2009). Using biological networks to search for interacting loci in genome-wide association studies.. Eur J Hum Genet.

[10] Bush WS, Dudek SM, Ritchie MD (2009). Biofilter: a knowledge-integration system for the multi-locus analysis of genome-wide association studies.. Pac Symp Biocomput.

[11] Schupbach T, Xenarios I, Bergmann S, Kapur K (2010). FastEpistasis: a high performance computing solution for quantitative trait epistasis.. Bioinformatics.

[12] Sinnott-Armstrong NA, Greene CS, Cancare F, Moore JH (2009). Accelerating epistasis analysis in human genetics with consumer graphics hardware.. BMC Res Notes.

[13] Zhang X, Pan F, Xie Y, Zou F, Wang W (2010). COE: a general approach for efficient genome-wide two-locus epistasis test in disease association study.. J Comput Biol.

[14] Stranger BE, Nica AC, Forrest MS, Dimas A, Bird CP (2007). Population genomics of human gene expression.. Nat Genet.

[15] Ronald J, Brem RB, Whittle J, Kruglyak L (2005). Local regulatory variation in Saccharomyces cerevisiae.. PLoS Genet.

[16] Lee SI, Dudley AM, Drubin D, Silver PA, Krogan NJ (2009). Learning a prior on regulatory potential from eQTL data.. PLoS Genet.

[17] Lee E, Bussemaker HJ (2010). Identifying the genetic determinants of transcription factor activity.. Mol Syst Biol.

[18] Zhu J, Zhang B, Smith EN, Drees B, Brem RB (2008). Integrating large-scale functional genomic data to dissect the complexity of yeast regulatory networks.. Nat Genet.

[19] Suthram S, Beyer A, Karp RM, Eldar Y, Ideker T (2008). eQED: an efficient method for interpreting eQTL associations using protein networks.. Mol Syst Biol.

[20] Evans DM, Marchini J, Morris AP, Cardon LR (2006). Two-stage two-locus models in genome-wide association.. PLoS Genet.

[21] Marchini J, Donnelly P, Cardon LR (2005). Genome-wide strategies for detecting multiple loci that influence complex diseases.. Nat Genet.

[22] Jensen LJ, Kuhn M, Stark M, Chaffron S, Creevey C (2009). STRING 8–a global view on proteins and their functional interactions in 630 organisms.. Nucleic Acids Res.

[23] Sun YV, Kardia SL (2010). Identification of epistatic effects using a protein-protein interaction database.. Hum Mol Genet.

[24] Dimas AS, Stranger BE, Beazley C, Finn RD, Ingle CE (2008). Modifier effects between regulatory and protein-coding variation.. PLoS Genet.

[25] Brem RB, Yvert G, Clinton R, Kruglyak L (2002). Genetic dissection of transcriptional regulation in budding yeast.. Science.

[26] Storey JD, Akey JM, Kruglyak L (2005). Multiple locus linkage analysis of genomewide expression in yeast.. PLoS Biol.

[27] Iafrate AJ, Feuk L, Rivera MN, Listewnik ML, Donahoe PK (2004). Detection of large-scale variation in the human genome.. Nat Genet.

[28] De Lobel L, Geurts P, Baele G, Castro-Giner F, Kogevinas M (2010). A screening methodology based on Random Forests to improve the detection of gene-gene interactions.. Eur J Hum Genet.

[29] Kellis M, Patterson N, Endrizzi M, Birren B, Lander ES (2003). Sequencing and comparison of yeast species to identify genes and regulatory elements.. Nature.

[30] Frazer KA, Ballinger DG, Cox DR, Hinds DA, Stuve LL (2007). A second generation human haplotype map of over 3.1 million SNPs.. Nature.

[31] Purcell S, Neale B, Todd-Brown K, Thomas L, Ferreira MA (2007). PLINK: a tool set for whole-genome association and population-based linkage analyses.. Am J Hum Genet.

