# Supplementary figures and images for: Comparison of Strategies to Detect Epistasis from eQTL Data
Source: PLoS One. 2011 Dec 19;6(12):e28415. doi: 10.1371/journal.pone.0028415 (PMC3242756; doi:10.1371/journal.pone.0028415)

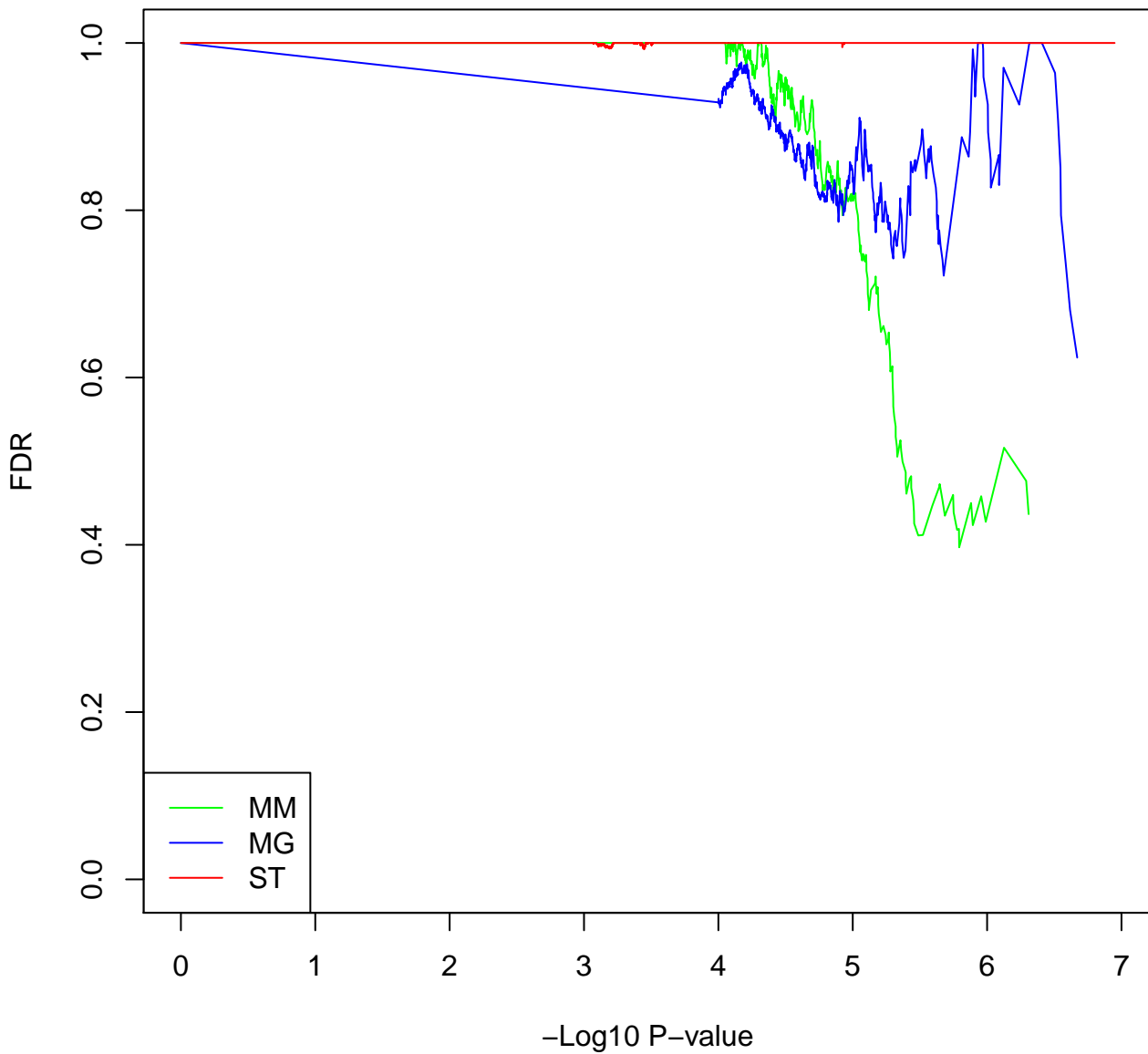

Supplement: Figure S1 — We plot the FDR for different p-value cutoffs, starting from the 0.1% quantile for the MM, MG and ST strategies separately. (PDF) [file pone.0028415.s001.pdf]

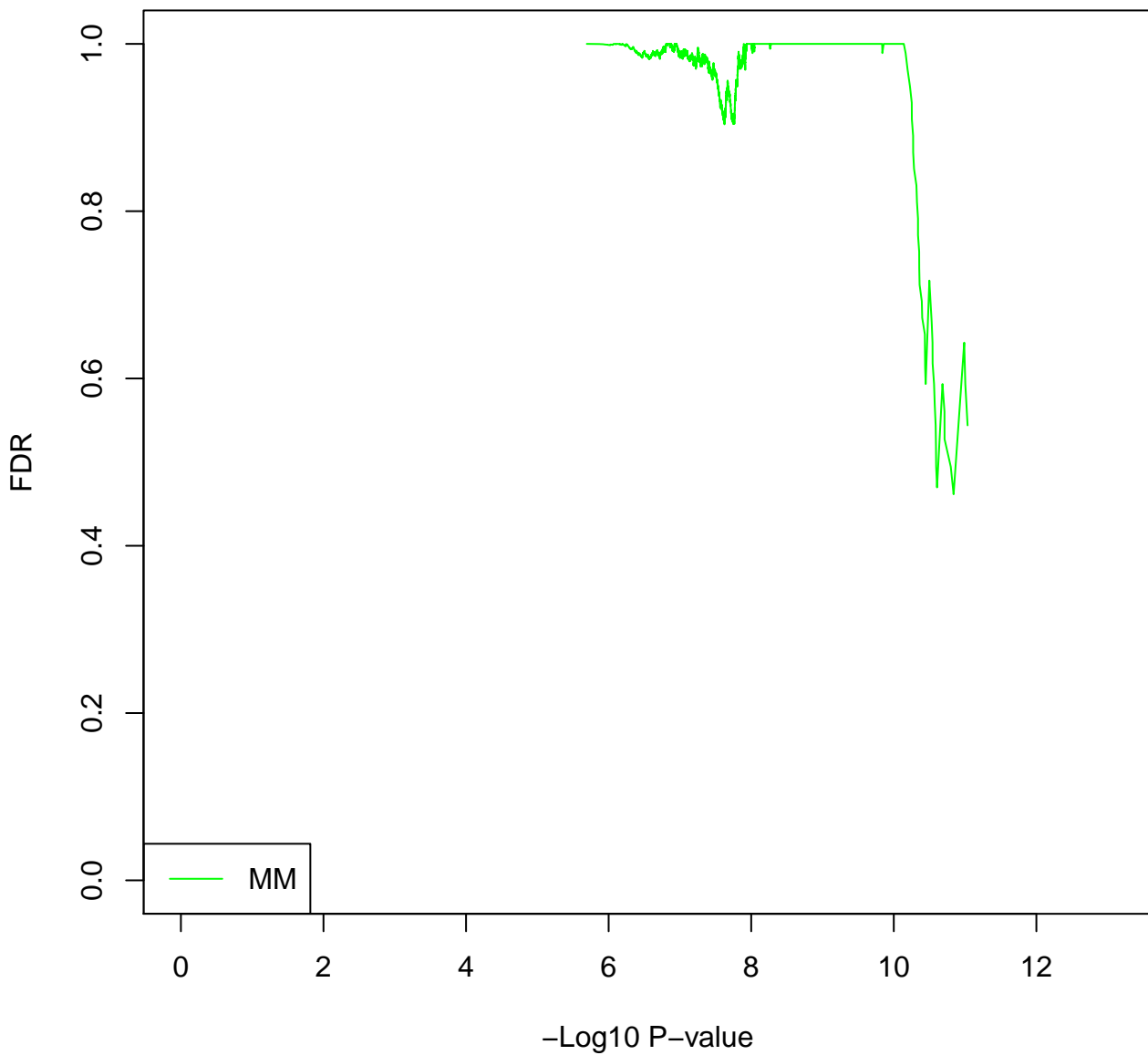

Supplement: Figure S3 — We plot the FDR results from the MM strategy (top 5000 marginally associated SNPs) for different p-value cutoffs, starting from the 0.1% quantile. (PDF) [file pone.0028415.s003.pdf]

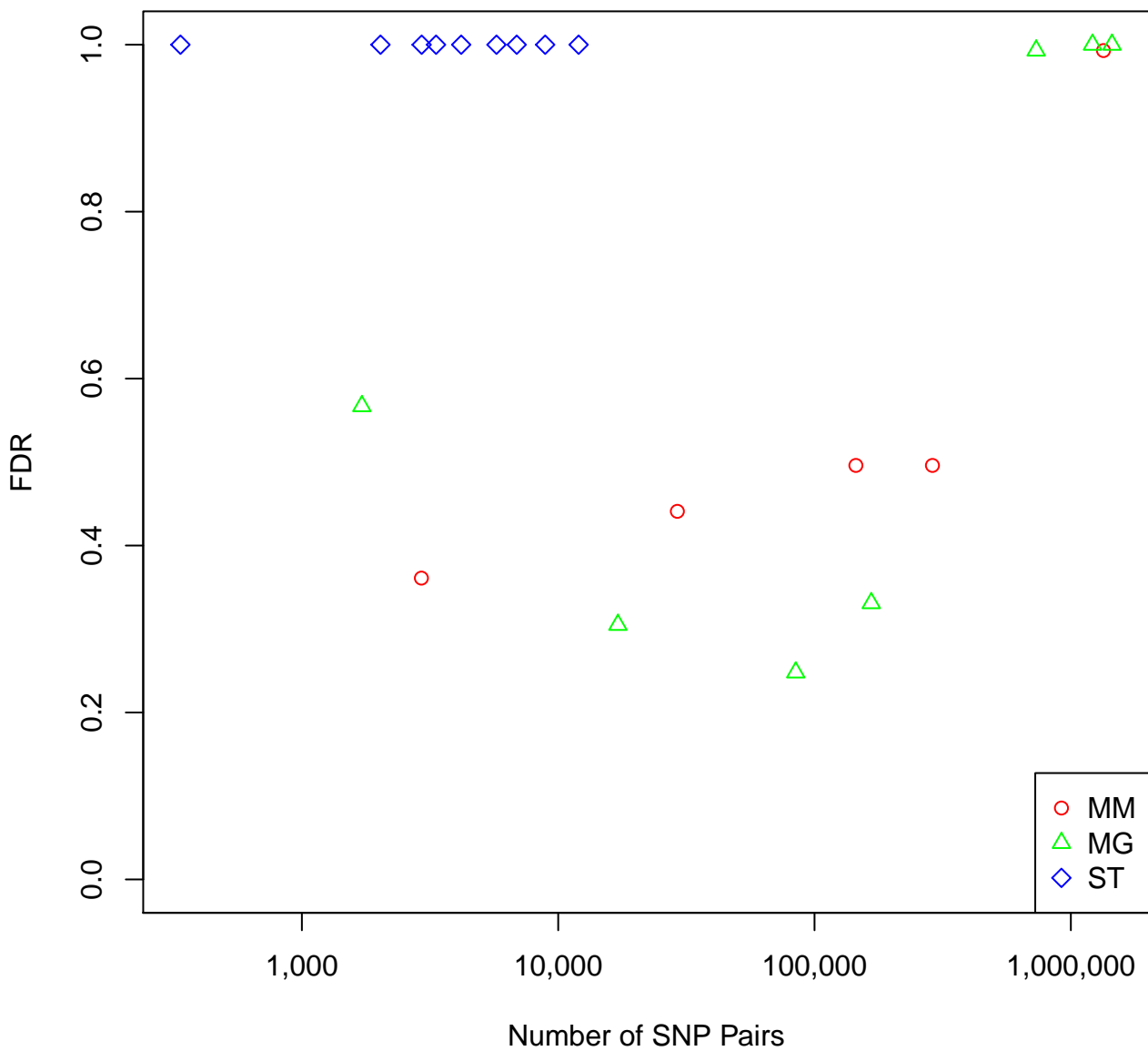

Supplement: Figure S5 — Comparison of the FDR (determined at cutoffs corresponding to the 0.1% quantile of permutation p-values) for detecting interactions in yeast gene expression data among the different subset strategies. The analysis is restricted to SNPs mapping to genes in STRING. The FDR is plotted against the number of SNP pairs for MM, MG and ST in red, green and blue, respectively. (PDF) [file pone.0028415.s005.pdf]
